# Acceptability and feasibility of policy implementation strategies for taxes earmarked for behavioral health services

**DOI:** 10.3389/frhs.2024.1304049

**Published:** 2024-04-04

**Authors:** Jonathan Purtle, Nicole A. Stadnick, Megan Wynecoop, Sarah C. Walker, Eric J. Bruns, Gregory A. Aarons

**Affiliations:** ^1^ Department of Public Health Policy & Management, Global Center for Implementation Science, New York University School of Global Public Health, New York, NY, United States; ^2^ Department of Psychiatry, Altman Clinical and Translational Research Institute Dissemination and Implementation Science Center, University of California San Diego, La Jolla, CA, United States; ^3^ Department of Psychiatry and Behavioral Sciences, University of Washington School of Medicine, Seattle, WA, United States

**Keywords:** policy, implementation science, acceptability, feasibility, behavioral health

## Abstract

**Background:**

This study's aims are to: (1) Compare the acceptability and feasibility of five types of implementation strategies that could be deployed to increase the reach of evidence-based practices (EBPs) with revenue from policies that earmark taxes for behavioral health services, and (2) Illustrate how definitions of implementation strategies and measures of acceptability and feasibility can be used in policy-focused implementation science research.

**Methods:**

Web-based surveys of public agency and community organization professionals involved with earmarked tax policy implementation were completed in 2022–2023 (*N* = 211, response rate = 24.9%). Respondents rated the acceptability and feasibility of five types of implementation strategies (dissemination, implementation process, integration, capacity-building, and scale-up). Aggregate acceptability and feasibility scores were calculated for each type of strategy (scoring range 4–20). Analyses of variance compared scores across strategies and between organizational actor types.

**Findings:**

For acceptability, capacity-building strategies had the highest rating (*M* = 16.3, SD = 3.0), significantly higher than each of the four other strategies, *p *≤ . 004), and scale-up strategies had the lowest rating (*M* = 15.6). For feasibility, dissemination strategies had the highest rating (*M* = 15.3, significantly higher than three of the other strategies, *p *≤ .002) and scale-up strategies had the lowest rating (*M* = 14.4).

**Conclusions:**

Capacity-building and dissemination strategies may be well-received and readily deployed by policy implementers to support EBPs implementation with revenue from taxes earmarked for behavioral health services. Adapting definitions of implementation strategies for policy-focused topics, and applying established measures of acceptability and feasibility to these strategies, demonstrates utility as an approach to advance research on policy-focused implementation strategies.

## Introduction

Although public policy has historically been understudied in the contemporary field of implementation science in health (1, 2), it has received increased attention in recent years (3–15). Conceptual frameworks for policy-focused work in the field have been developed (16, 17) and reviews (18–20) have identified measures to characterize policy implementation processes and describe how policy functions as an outer-setting determinant of the delivery of clinical interventions. Despite these advances, research and scholarship on strategies to support policy implementation remains underdeveloped.

While several implementation strategies in the Expert Recommendations for Implementing Change (ERIC) compendium involve policies (e.g., “provide access to new funding,” “mandate change”) (21), these strategies emphasize the implementation of clinical evidence-based interventions—not the policy itself. Some qualitative work has used ERIC constructs to code strategies used to support policy implementation (22, 23), but the implementation science literature provides little guidance about how to generate evidence to inform decisions about the types of strategies perceived to be most relevant to a particular policy implementation context.

It is well-established that clinically-focused implementation strategies should be perceived as acceptable and feasible to the professionals who would use them (24–26). However, virtually no prior work has quantitatively assessed the acceptability or feasibility of strategies to support policy implementation. This Brief Research Report presents results of an exploratory study of the perceived acceptability and feasibility of potential strategies to support policy implementation. The Report also provides a methodological case example of how acceptability and feasibility can be assessed in a policy implementation study.

### Policies that earmarked taxes for behavioral health services

The current study focuses on the implementation of state and local governmental policies that earmark tax revenue for behavioral health (i.e., mental health and substance use disorder) services in the United States (27). Detailed descriptions of these tax policies and the larger policy implementation study from which data are drawn are provided elsewhere (27–31). In short, an earmarked tax is one placed on a specific base (e.g., goods, property, income) for which revenue is dedicated to a specific purpose (32–34). As of 2022, a legal mapping study found that there were at least 207 policies in the United States that earmark tax revenue for behavioral health services and that the number of jurisdictions adopting these policies has increased drastically over the past two decades (30). These taxes generate a substantial amount of revenue, about $3.57 billion annually, and approximately 30% of the U.S. population lives in a jurisdiction with such a tax (30).

Through the creation of a new sustainable and dedicated source of funding, these earmarked tax policies have potential to enhance the reach (i.e., number of people served) of EBPs and the fidelity with which they are implemented (27–31, 35, 36). Professionals involved with earmarked tax policy implementation report many benefits to the financing approach (31), yet these taxes do not necessarily increase the reach of EBPs. For example, a survey of 155 professionals involved with earmarked tax policy implementation in California and Washington found that only about two-thirds strongly agreed that the tax policies increased the number of people served by behavioral health EBPs (31). Although supporting EBP implementation is just one possible goal of earmarked taxes, policy implementation strategies have potential to help achieve this goal. Assessing the acceptability and feasibility of implementation strategies in this policy context is a first step towards candidate strategies that could be deployed at scale, and evaluated in future research.

### Study aims

To develop an evidence base related to implementation strategies for policies that earmark tax revenue for behavioral health, and to advance work on policy implementation strategies more broadly, the aims of this study are to:

1. Compare perceptions of the acceptability and feasibility of five types of strategies that could be deployed to support EBP implementation with revenue from policies that earmark taxes for behavioral health services; and
2. Illustrate how definitions of types of implementation strategies were adapted for survey questions focused on policy implementation and demonstrated how measures of acceptability and feasibility were used to assess perceptions of these strategies in a policy implementation context.

## Method

### Sample and data collection

The methods for the larger policy implementation study are detailed in the published study protocol (27). The study was approved by the MASKED Institutional Review Board (27). The data presented here come from web-based surveys of government and community organization professionals involved with oversight, decision making, and implementation policies which earmark taxes for behavioral health services. These professionals were in positions such as, but not limited to, tax coordinators, leaders of state and county behavioral health agencies, and members of county tax advisory boards. Jurisdictions with policies that earmarked taxes for behavioral health were identified through the aforementioned legal mapping study (30). The survey sample frame was created of professionals involved with earmarked tax policy implementation in seven states: California, Washington, Ohio, Illinois, Missouri, Colorado, and Kansas. The sample frame was created from contact databases maintained by practice partners (e.g., state and county behavioral health professional associations), internet searches, and databases of behavioral health officials compiled by the research team for prior studies (37–39).

Web-based surveys were e-mailed to professionals involved with earmarked tax policy implementation between September 2022 and May 2023. Up to eight personalized e-mails were sent with a unique survey link, and telephone follow-up was conducted. To capture the perspectives of professionals involved with earmarked tax policy implementation who were not included in the original sample frame, we also created an open (i.e., not unique) survey link that was circulated by our aforementioned practice partners. A $20 gift card for survey completion was offered. All four questions about the acceptability or feasibility of at least one implementation strategy (detailed below) were completed by 211 respondents. The response rate for the unique link surveys was 24.9%, consistent with recent state-wide surveys of behavioral health officials (37–39), and 81.1% of responses were from unique survey links (as opposed to the open survey link). The distribution of respondents across states was: California = 35.4%, Washington = 25.0%, Ohio = 21.7%, Illinois = 7.5%, Colorado = 5.2%, Missouri = 4.7%, Kansas = 0.5%. This distribution reflects the number of counties in each state involved with implementing an earmarked tax.

### Measures

The survey questions and format are included as a Supplementary. In the survey, respondents were separately presented with adapted definitions of Leeman et al.'s five types of implementation strategies: dissemination, implementation process, integration, capacity-building, and scale-up (40). The Leeman et al.’ typology of strategies was derived from Powell et al.'s ERIC compendium (21). Definitions in the survey were adapted for the earmarked tax policy implementation context using Proctor et al.'s recommendations for specifying implementation strategies (41). The strategy *actor*, *action*, and *action target* (i.e., who or what was the intended target) were all anchored to the broad *implementation outcome* of earmarked tax policy revenue supporting the implementation of EBPs. Table 1 shows the definitions of each strategy and actor type that were provided in the survey.

**Table 1:** Definitions of leeman et al.'s of types of implementation strategies and actor types, adapted to focus on policies that earmark taxes for behavioral health services achieving the outcome of increasing the reach of evidence-based practices.

Construct	Wording of definition in survey^a^
Policy implementation strategy type	
Dissemination strategies	These strategies entail your organization **communicating information** to behavioral health service organizations to increase leaders’ and providers’ knowledge and improve their attitudes about evidence-based practices that can be funded with earmarked behavioral health tax revenue.
Implementation process strategies	These strategies entail your organization **helping behavioral health service organizations’ select** evidence-based practices funded by earmarked behavioral health tax revenue, plan for their integration, and **evaluate** their impacts.
Integration strategies	These strategies entail your organization **changing the organizational context** within behavioral health service organizations to ensure the delivery of evidence-based practices funded by earmarked behavioral health tax revenue (e.g., by using clinical reminder systems, quality monitoring activities, and changing professional roles with organizations).
Capacity-building strategies	These strategies entail your organization **increasing the capacity** of behavioral health service organizations to select and integrate evidence-based practices funded by earmarked behavioral health tax revenue and evaluate their impacts (e.g., by enhancing the motivation and self-efficacy of leadership and direct service providers).
Scale-up strategies	These strategies entail your organization **increasing the ability** of behavioral health service organizations to ensure that evidence-based practices funded by earmarked behavioral health tax revenue achieve desired outcomes (e.g., by providing training on evidence-based practice to direct service providers).
Policy implementation actor type	
Delivery system actors	Providing direct behavioral health and social services with tax revenue
Support system actors	Supporting system and capacity building efforts for organizations that provide direct behavioral health and social services with tax revenue
Synthesis and translation system actors	Reviewing evidence about promising approaches to using earmarked tax revenue and communicating this information to organizations that provide direct behavioral health and social services

^a^ Bolded emphasis included in survey.

With the definition of each strategy separately displayed on a single web-based survey screen, respondents rated the acceptability and feasibility of each type of implementation strategy in terms of it being used by their organization to support the implementation of EBPs with earmarked tax revenue. Acceptability is defined as the perception a category of implementation strategy is agreeable, palatable, or satisfactory; whereas feasibility is defined as the extent to which a category of implementation strategy can be successfully used or carried out within a given agency or setting (25). These constructs were assessed using Weiner et al.'s measures of acceptability (four items, *α* = .85) and feasibility (four items, *α* = .89) (25). Reponses were summed to calculate aggregate acceptability and feasibility scores for each type of policy implementation strategy (possible scoring range 4–20 for each measure).

Next, respondents separately indicated all of the “actor types”—derived from Leeman et al.'s typology of organizations that can use implementation strategies—that accurately characterized all of their organization's role in earmarked tax policy implementation. Definitions of each of the actor types (i.e., delivery system actors, support system actors, synthesis and translation system actors) was provided, with wording adapted to be focused on their organization's role in earmarked tax policy implementation. Respondents were instructed to select all of the actor types that applied. The proportion of respondents endorsing each actor type was: delivery system actors 52.1%, support system actors 74.2%, and synthesis and translation system actors 40.7%.

### Analysis

Cronbach's alpha was calculated for the acceptability and feasibility ratings of each policy implementation strategy. Descriptive statistics (i.e., means, standard deviations, skewness) were calculated for all acceptability and feasibility ratings. Bivariate correlations between the acceptability and feasibility ratings of each strategy were assessed. Two-tailed, paired sample *t*-tests assessed the statistical significance of differences in acceptability and feasibility ratings, respectively, across the implementation strategies. Separate ANOVAs compared differences in acceptability and feasibility ratings between respondents who characterized their organization according to different actor types.

## Results

Table 2 shows descriptive statistics for the acceptability and feasibility each policy implementation strategy. For each of the five strategies, measures of acceptability (*α* range = 86–.91) and feasibility (*α* range = .94–.97) demonstrated strong internal consistency. The mean policy implementation strategy acceptability rating was highest for capacity building strategies (mean = 16.3, SD = 3.0) and lowest for scale-up strategies (mean = 15.6, SD = 3.4). The mean policy implementation strategy feasibility rating was highest for dissemination strategies (mean = 15.3, SD = 3.2) and lowest for scale-up strategies (mean = 14.4, SD = 3.7).

**Table 2:** Descriptive statistics of ratings of acceptability and feasibility of implementation strategies intended to increase the reach of evidence-based practices with revenue from policies that earmark taxes for behavioral health services.

Strategy	Acceptability					Feasibility				
	*n*	Mean	SD	Skew	*α*	*n*	Mean	SD	Skew	α
Dissemination strategies	195	15.9	3.0	−0.1	0.86	197	15.3	3.2	−0.1	0.94
Implementation process strategies	190	15.9	3.2	−0.5	0.91	190	15.1	3.6	−0.3	0.96
Integration strategies	181	15.7	3.3	−0.4	0.91	184	14.5	3.7	−0.2	0.97
Capacity-building strategies	200	16.3	3.0	−0.5	0.87	199	14.7	3.6	−0.2	0.94
Scale-up strategies	191	15.6	3.4	−0.5	0.91	192	14.4	3.7	−0.2	0.96

Possible scoring range 4–20.

**Figure 1:**
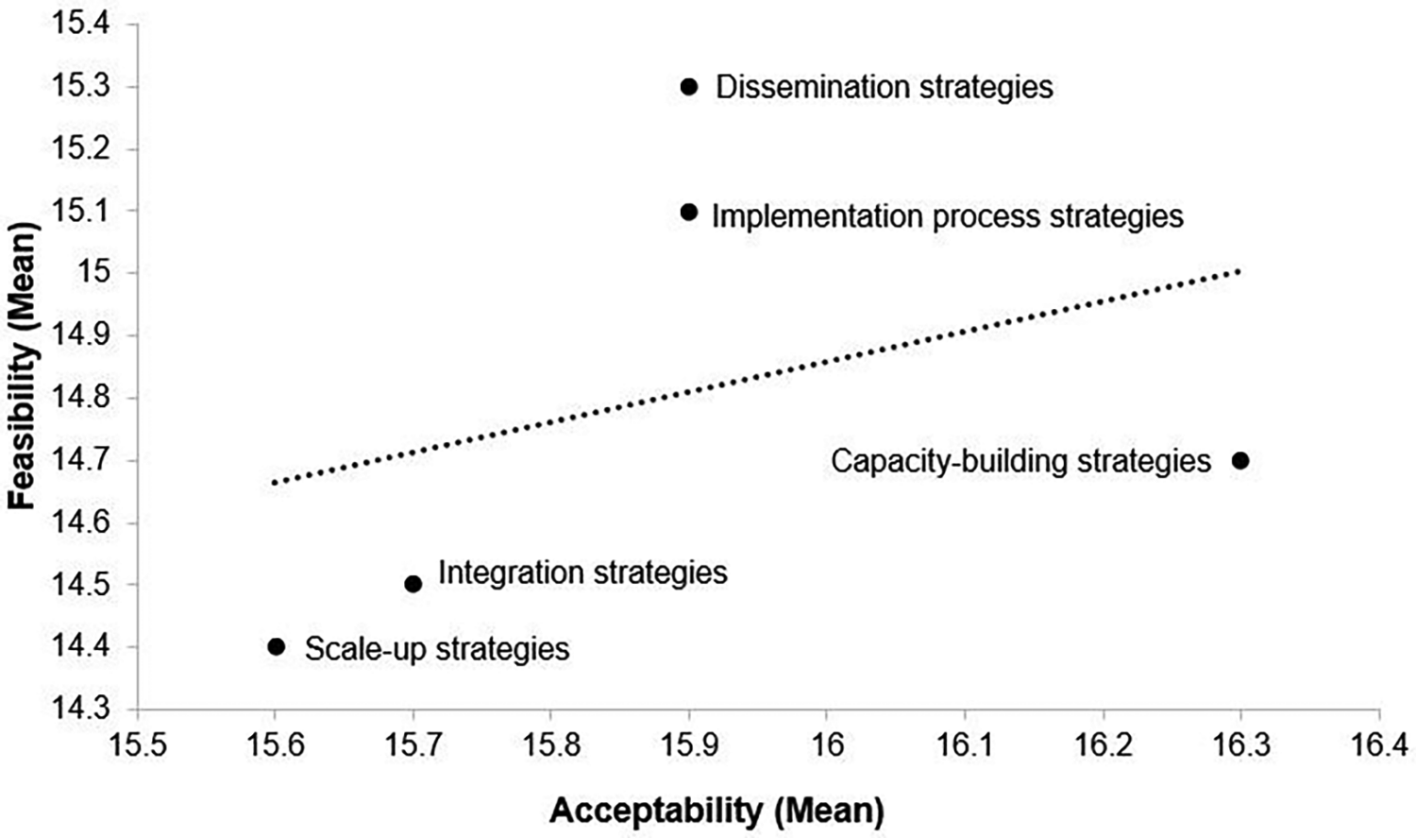
Plot of mean ratings of acceptability and feasibility of implementation strategies intended to increase the reach of evidence-based practices with revenue from policies that earmark taxes for behavioral health services. Possible scoring range 4–20.

Figure 1 plots the mean acceptability and feasibility ratings for each strategy. As shown, scale-up and integration strategies were rated as least acceptable and least feasible. For each strategy, there was a statistically significant (*p *< .001) positive correlation between ratings of acceptability and feasibility. The mean Pearson correlation coefficient for the five strategies was 0.72 and the magnitude of correlations ranged from 0.81 for dissemination strategies to 0.50 for capacity building strategies.

Table 3 shows the effect sizes and statistical significance of pairwise comparisons between mean ratings of each type of policy implementation strategy. For acceptability, capacity building strategies were rated as significantly (*p *≤ .008) more acceptable than all four other strategies (e.g., *p *< .001 Cohen's D = −0.26 for capacity building strategies vs. integration strategies). For feasibility, dissemination strategies were rated as significantly (*p *≤ .001) more feasible than integration, capacity building, and scale-up strategies. (e.g., *p *< .001 Cohen's D = 0.33 for dissemination strategies vs. scale-up strategies). There were no significant differences in acceptability or feasibility ratings when compared between respondent who classified their organization according to different actor types.

**Table 3:** Significance of pairwise comparisons of the acceptability and feasibility of implementation strategies intended to increase the reach of evidence-based practices with revenue from policies that earmark taxes for behavioral health services.

Implementation strategies compared	*P*-value	Cohen's d
Acceptability		
Dissemination vs. implementation process	.74	−0.02
Dissemination vs. integration	.13	0.11
Dissemination vs. capacity-building	.007	−0.20
Dissemination vs. scale-up	.39	0.06
Implementation process vs. integration	.09	0.13
Implementation process vs. capacity-building	.008	−0.20
Implementation process vs. scale-up	.22	0.09
Integration vs. capacity-building	<.001	−0.26
Integration vs. scale-up	1.000	0.00
Capacity-building vs. scale-up	<.001	0.37
Feasibility		
Dissemination vs. implementation process	.05	0.14
Dissemination vs. integration	<.001	0.37
Dissemination vs. capacity-building	.001	0.24
Dissemination vs. scale-up	<.001	0.33
Implementation process vs. integration	<.001	0.29
Implementation process vs. capacity-building	0.12	0.12
Implementation process vs. scale-up	0.002	0.24
Integration vs. capacity-building	0.20	−0.10
Integration vs. scale-up	0.52	0.05
Capacity-building vs. scale-up	0.03	0.16

## Discussion

This study presents a quantitative assessment of the acceptability and feasibility of policy implementation strategies. Results shed light on the types of strategies that policy actors judged to be feasible and acceptable to deploy to support the implementation of EBPs with revenue from policies that earmark taxes for behavioral health services. Capacity-building strategies were perceived as the most acceptable strategy to support the implementation of EBPs through these policies, whereas scale-up strategies were identified as least acceptable (as well as least feasible). Although capacity building and scale-up strategies both target the skills and motivation of service providers and organizational leaders, capacity building strategies—as defined in the survey—afford more autonomy to service organizations in terms of selecting EBPs. Scale-up strategies, in contrast, focus on “ensuring” that EBPs funded by tax revenue “achieve desired outcomes.” It is possible that this prescriptive language was not well-received by respondents and contributed to lower ratings of acceptability (42).

The finding that dissemination strategies were perceived as most feasible is not surprising given that asynchronous communication of information is typically not resource intensive or politically contentious (43). It is promising that organizations involved with the implementation of earmarked tax policies find dissemination strategies feasible, as well as acceptable, because responsibilities for dissemination are often unspecified in research translation pipelines (44, 45). Dissemination strategies are understudied in implementation science (46, 47), however, and research is needed to inform how organizations might develop messages that are effective at promoting the use of earmarked tax revenue to support EBP delivery.

The methods describe in this Research Brief Report illustrate how definitions of implementation strategies can be adapted for a survey focused on policy implementation. Furthermore, the Report demonstrates how widely used and pragmatic measures of acceptability and feasibility can used in policy implementation research. Weiner et al.'s measures of acceptability and feasible demonstrated strong internal consistency when used to assess policy implementation strategies. However, minimal variance between ratings of these strategies raises questions about their suitability. More in-depth psychometric testing of these measures' applicability to policy implementation strategies is warranted in future research.

### Limitations

Findings should be considered within the context of the study's limitations. First, although we observed statistically significant differences in ratings of the acceptability and feasibility of policy implementation strategies, the practical significance of these differences are unclear. Mean ratings of acceptability and feasibility across all strategies were consistently high (i.e., mean ≥14.4 on 20-point scale), suggesting that none of these strategies were perceived as unacceptable or infeasible. The average effect size (Cohen's D) of statiscally significant differences between pairwise ratings of strategies was only 0.13. Relatedly, while acceptability and feasibility are considered conceptually distinct constructs in the field of implementation science research, the extent to which they were perceived as distinct by respondents is uncertain. The fact that there was a statistically significant correlation between the rating of acceptability and feasibility for each strategy suggests that respondents may not have perceived the two constructs of conceptually distinct.

Second, although the response rate of 24.9% is consistent with recent state-wide surveys of behavioral health officials (37–39), respondents may not fully reflect the perspectives of all professionals involved with behavioral health earmarked tax policy implementation. Third, definitions of all strategies were anchored to the broad policy implementation outcome of earmarked tax revenue supporting the implementation of EBPs. As noted, supporting the implementation of EBPs is just one possible goal of policies that earmark taxes for behavioral health. Interviews conducted as part of the larger policy implementation study (27) have revealed that other outcomes—such as reducing inequities in access to behavioral health services and enhancing service infrastructure—are often primary goals of the taxes. Ratings of implementation strategy acceptability and feasibility may have varied if definitions were anchored to a different policy implementation outcome. Fourth, it should be emphasized that the study focused on perceptions of the acceptability and feasibility of implementation strategies and does not shed light on the extent to which these strategies may be effective at supporting EBP implementation. Finally, the survey did not assess if, or the extent to which, respondents had actually used the implementation strategies they rated. Experiences using the strategies would likely affect rating of acceptability and feasibility.

## Conclusions

Within the context of the implementation of policies that earmark taxes for behavioral health services, capacity building strategies and dissemination strategies may be well-received and deployed by organizations involved with tax policy implementation to support the implementation of EBPs. Adapting definitions of implementation strategies for policy-focused topics, and using established measures of acceptability and feasibility to elicit feedback about these strategies, demonstrates utility as an approach to advance research of policy-focused implementation strategies.

## Data Availability

The raw data supporting the conclusions of this article will be made available by the authors, without undue reservation.
