# Supplementary material for: Acceptability and feasibility of policy implementation strategies for taxes earmarked for behavioral health services
Source: Front Health Serv. 2024 Apr 4;4:1304049. doi: 10.3389/frhs.2024.1304049 (PMC11025354; doi:10.3389/frhs.2024.1304049)
Supplement: Supplementary file 1 [file Datasheet1.docx]

*Last are questions about your perceptions of five different types of implementation strategies that your organization could use to maximize the  benefits of the earmarked tax for behavioral health in your jurisdiction.*

**Dissemination strategies:** These strategies entail your organization **communicating information** to behavioral health service organizations to increase leaders and providers knowledge and improve their attitudes about evidence-based practices that can be funded with earmarked behavioral health tax revenue.

|  | **1= Completely disagree** | **Completely agree= 5** | Not Applicable |
| --- | --- | --- | --- |

|  | 1 | 2 | 3 | 4 | 5 |
| --- | --- | --- | --- | --- | --- |

| Dissemination strategies meet my approval () | 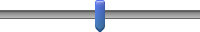 |
| --- | --- |
| Dissemination strategies are appealing to me () | 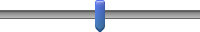 |
| I like dissemination strategies () | 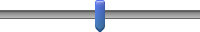 |
| I welcome dissemination strategies () | 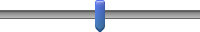 |
| Dissemination strategies seem implementable () | 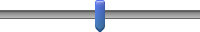 |
| Dissemination strategies seem possible () | 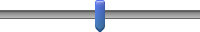 |
| Dissemination strategies seem doable () | 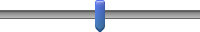 |
| Dissemination strategies seem easy to use () | 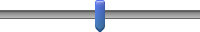 |

**Implementation process strategies:** These strategies entail your organization **helping behavioral health service organizations’ select** evidence-based practices funded by earmarked behavioral health tax revenue, plan for their integration, and **evaluate** their impacts.

|  | **1= Completely disagree** | **Completely agree= 5** | Not Applicable |
| --- | --- | --- | --- |

|  | 1 | 2 | 3 | 4 | 5 |
| --- | --- | --- | --- | --- | --- |

| Implementation process strategies meet my approval () | 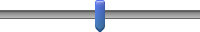 |
| --- | --- |
| Implementation process strategies are appealing to me () | 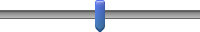 |
| I like implementation process strategies () | 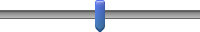 |
| I welcome implementation process strategies () | 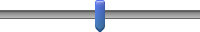 |
| Implementation process strategies seem implementable () | 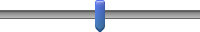 |
| Implementation process strategies seem possible () | 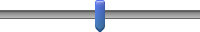 |
| Implementation process strategies seem doable () | 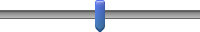 |
| Implementation process strategies seem easy to use () | 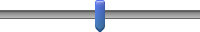 |

**Integration strategies:** These strategies entail your organization **changing the organizational context** within behavioral health service organizations to ensure the delivery of evidence-based practices funded by earmarked behavioral health tax revenue (e.g., by using clinical reminder systems, quality monitoring activities, and changing professional roles with organizations).

|  | **1= Completely disagree** | **Completely agree= 5** | Not Applicable |
| --- | --- | --- | --- |

|  | 1 | 2 | 3 | 4 | 5 |
| --- | --- | --- | --- | --- | --- |

| Integration strategies meet my approval () | 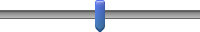 |
| --- | --- |
| Integration strategies are appealing to me () | 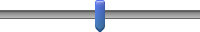 |
| I like integration strategies () | 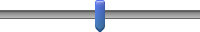 |
| I welcome integration strategies () | 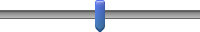 |
| Integration strategies seem implementable () | 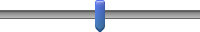 |
| Integration strategies seem possible () | 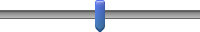 |
| Integration strategies seem doable () | 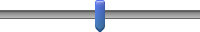 |
| Integration strategies seem easy to use () | 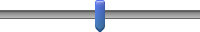 |

Q18  **Capacity-building strategies**: These strategies entail your organization **increasing the capacity** of behavioral health service organizations to select and integrate evidence-based practices funded by earmarked behavioral health tax revenue and evaluate their impacts (e.g., by enhancing the motivation and self-efficacy of leadership and direct service providers).

|  | **1= Completely disagree** | **Completely agree= 5** | Not Applicable |
| --- | --- | --- | --- |

|  | 1 | 2 | 3 | 4 | 5 |
| --- | --- | --- | --- | --- | --- |

| Capacity-building strategies meet my approval () | 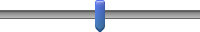 |
| --- | --- |
| Capacity-building strategies are appealing to me () | 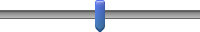 |
| I like capacity-building strategies () | 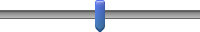 |
| I welcome capacity-building strategies () | 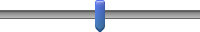 |
| Capacity-building strategies seem implementable () | 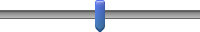 |
| Capacity-building strategies seem possible () | 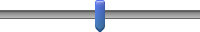 |
| Capacity-building strategies seem doable () | 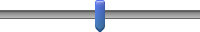 |
| Capacity-building strategies seem easy to use () | 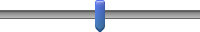 |

Q19  **Scale-up strategies:** These strategies entail your organization **increasing the ability** of behavioral health service organizations to ensure that evidence-based practices funded by earmarked behavioral health tax revenue achieve desired outcomes (e.g., by providing training on evidence-based practice to direct service providers).

|  | **1= Completely disagree** | **Completely agree= 5** | Not Applicable |
| --- | --- | --- | --- |

|  | 1 | 2 | 3 | 4 | 5 |
| --- | --- | --- | --- | --- | --- |

| Scale-up strategies meet my approval () | 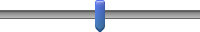 |
| --- | --- |
| Scale-up strategies are appealing to me () | 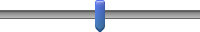 |
| I like scale-up strategies () | 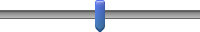 |
| I welcome scale-up strategies () | 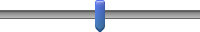 |
| Scale-up strategies seem implementable () | 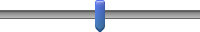 |
| Scale-up strategies seem possible () | 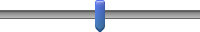 |
| Scale-up strategies seem doable () | 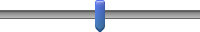 |
| Scale-up strategies seem easy to use () | 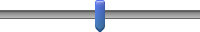 |

| 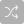 |
| --- |

Q3 Please indicate which categories most accurately describe **your organization’s** role within the context of implementing the earmarked tax for behavioral health in your jurisdiction. Select all that apply.

- Providing direct behavioral health and social services with tax revenue (1)
- Supporting system and capacity building efforts for organizations that provide direct behavioral health and social services with tax revenue (2)
- Reviewing evidence about promising approaches to using earmarked tax revenue and communicating this information to organizations that provide direct behavioral health and social services (3)
